# Self-reported side effects of the Oxford AstraZeneca COVID-19 vaccine among healthcare workers in Ethiopia, Africa: A cross-sectional study

**DOI:** 10.3389/fpubh.2022.937794

**Published:** 2022-07-19

**Authors:** Elias Ali Yesuf, Abanoub Riad, Ahmad Sofi-Mahmudi, Morankar Sudhakar, Addisalem Mekonnen, Selamawit Endalkachew, Feyissa Mama, Semira Muhidin, Bethelhem Ayele, Mohammed Yahya, Abduselam Usman, Jemal Abafita, Miloslav Klugar

**Affiliations:** ^1^Department of Health Policy and Management, Jimma University, Jimma, Ethiopia; ^2^Department of Public Health, Masaryk University, Brno, Czechia; ^3^Czech National Center for Evidence-Based Healthcare and Knowledge Translation (Cochrane Czech Republic, Czech EBHC: JBI Center of Excellence, Masaryk University GRADE Center), Institute of Biostatistics and Analyses, Masaryk University, Brno, Czechia; ^4^Cochrane Iran Associate Center, National Institute for Medical Research Development, Tehran, Iran; ^5^Seqiz Health Network, Kurdistan University of Medical Sciences, Seqiz, Iran; ^6^Department of Health, Behavior and Society, Jimma University, Jimma, Ethiopia; ^7^Jimma University Medical Center, Jimma University, Jimma, Ethiopia; ^8^Department of Pediatrics and Child Health, Jimma University, Jimma, Ethiopia; ^9^Department of Public Health, Woldia University, Woldia, Ethiopia; ^10^Gebretsadik Shawo Hospital, Southwest Health Bureau, Bonga, Ethiopia; ^11^Jigjiga University Specialized Hospital, Jigjiga University, Jigjiga, Ethiopia; ^12^Department of Gynecology and Obstetrics, Jigjiga University, Jigjiga, Ethiopia; ^13^Department of Economics, Jimma University, Jimma, Ethiopia; ^14^Institute of Health Information and Statistics of the Czech Republic, Prague, Czechia

**Keywords:** Healthcare workers, COVID-19 vaccine, side effects, Oxford AstraZeneca, Ethiopia, Africa

## Abstract

**Introduction:**

Ethiopia is the second most populous country in Africa. Ethiopia received most of its COVID-19 vaccines through donations. The Oxford AstraZeneca vaccine is the first to be donated to Ethiopia by the COVAX facility. Healthcare workers were the priority population that received the Oxford AstraZeneca COVID-19 vaccine. However, there was no nationwide study on the safety of the vaccine in Ethiopia. This study aimed to measure the prevalence and predictors of self-reported side effects of the Oxford AstraZeneca vaccine.

**Materials and methods:**

The study employed a cross-sectional design. A sample of healthcare workers who took Oxford AstraZeneca COVID-19 vaccine was drawn from four regions of Ethiopia; namely, Amhara, Oromia, Somali, and Southwest. Data were collected on sociodemographic characteristics, medical anamnesis, COVID-19 related anamnesis, and COVID-19 vaccine anamnesis *via* telephone interview. Descriptive and inferential analyses were done. The software, IBM SPSS Statistics v21.0, was used for analyses of data.

**Results:**

Out of 384 people, 346 responded (response rate: 90.1%). Female accounted for 34.1% of the respondents. The mean age of the respondents was 31.0 years (Standard Deviation (SD) = 7.4). Nurses accounted for 43.7% of the respondents. The prevalence of at least one local- and systemic-side effect was 50.6 and 44.5%, respectively. The most frequent local- and systemic- side effect were injection site pain and headache, respectively. Both types of side effects mostly subsided in the first 3 days. A third of healthcare workers with side effects took at least one medication. Paracetamol followed by diclofenac sodium were taken by healthcare workers to overcome side effects. There was no independent predictor of local side effect. After controlling for age and chronic diseases, the odds of healthcare workers with COVID-19 like symptoms to experience systemic side effects was 1.38 (Confidence Interval (CI): 1.04–1.82) times more than that of healthcare workers without COVID-19 like symptoms.

**Conclusions:**

The prevalence of local- and systemic-side effects of the Oxford AstraZeneca COVID-19 vaccine was modest. As the symptoms were mostly common in the first 3 days, it is preferable to monitor healthcare workers at least in the first 3 days following the administration of the vaccine.

## Introduction

Coronavirus disease 19 (COVID-19) started as a local outbreak in Wuhan, China and subsequently spread all over the world becoming a pandemic. It resulted in millions of deaths across the world (1).

Several COVID-19 control strategies were devised. The strategies are broadly classified as non-pharmacological interventions, vaccines, and treatment. Social distancing is one of the most effective interventions (2). For Low- and Middle-Income Countries (LMICs), generalized lockdown, zonal lockdown, and rolling lockdown are recommended depending on the epidemiologic, economic, and health system capabilities (3).

While some drug treatments for COVID-19 have been suggested (4), vaccines which were introduced in the second year of the pandemic seem to be the most effective treatments against the disease to date (4). There are five types of vaccines, such as live attenuated, inactivated, protein-based, nucleic acid, and viral vector (5). Few of the COVID-19 vaccines approved through the Emergency Use Authorization (EUA) include Pfizer/BioN-Tech, Moderna, Oxford Astra-Zeneca, Sputnik V, Covaxin, and Sinovac. United States of America, United Kingdom, Russia, India, and China are the major countries which have granted authorization for the vaccines (5).

Before authorization by relevant authorities, vaccines undergo rigorous studies in phase I and phase II clinical trials to determine their efficacy and safety. One particular safety concern is vaccine-related immunopathology that occurs in vaccinated people during a natural infection (6). Once COVID-19 vaccines are found to be efficacious and safe during clinical trials, then they will be deployed. However, the safety of vaccines after deployment should be studied. Several tools can be used to study the safety of COVID-19 vaccines. A few of the tools are active surveillance and passive surveillance (7).

In countries, such as Ethiopia passive surveillance using administrative data is not feasible because of resource limitations. In order to overcome this limitation, active surveillance using cross-sectional studies is helpful.

Ethiopia received most of its vaccines through donations. Two million two hundred thousand million doses of Oxford Astra Zeneca COVID-19 vaccine were the first batch (27) and were received from the COVAX facility –which is an international partnership aimed at supplying vaccines to lower income countries. Subsequently China donated 1.8 million doses of the Sinopharm COVID-19 vaccine with the aim of improving vaccine accessibility (8). Finally, the United States donated nearly two million doses of the Pfizer COVID-19 vaccine to Ethiopia (9). Despite all these efforts, the percentage of the population which received COVID-19 vaccines is still very low.

Priority was given to healthcare providers to receive vaccine shots followed by elderly and people with co-morbidities. When the vaccines were rolled-out in Ethiopia on March 13, 2021 with Oxford AstraZeneca COVID-19 vaccine, most healthcare workers received the Oxford AstraZeneca COVID-19 vaccine because it was the first vaccine shipped to Ethiopia in large quantities.

According to some studies, in Ethiopia, half of healthcare workers (10) and a third of the general population are willing to accept COVID-19 vaccines (11).

In Low- and Middle-Income Countries (LMIC) compared with high income a higher mean acceptance rate of 80% was reported for COVID-19 vaccines (12). For example, vaccine acceptance among university students in a high income country, such as the Czech Republic was 73.3% (13). Similar rates of hesitancy were observed among healthcare workers in Arab countries (14). Perceived vaccine safety helps to increase vaccine acceptance. Vaccine hesitancy was negatively associated with willingness to accept COVID-19 vaccines (10) including booster doses (13).

It is important to study the safety of vaccines to improve acceptance and increase inoculation percentages. Studies reported the prevalence of adverse effects in Ethiopia. One study undertaken in South Ethiopia found a 44% prevalence of local pain and a 40% prevalence of fever. However, this study focused on one region (15). Another study from Ethiopia reported 65% prevalence of injection site pain and 50% prevalence of headache (16). Nevertheless, this study did not report the determinants of the side effects. Therefore, there is a need for a national study on the prevalence and determinants of side effects of the Oxford AstraZeneca COVID-19 vaccine.

The study was aimed at measuring the prevalence of self-reported side effects of the Oxford AstraZeneca COVID-19 vaccine among health care workers in Ethiopia.

## Materials and methods

### Oxford AstraZeneca COVID-19 vaccine

The Oxford AstraZeneca vaccine was developed by the University of Oxford. It has SARS CoV-2 surface protein (nCoV-19) in a vector from chimpanzee adenovirus (ChAdOx1) (17). It's route of administration is intramuscular.

### Design

A cross-sectional study design was conducted and reported according to the Strengthening the Reporting of Observational Studies in Epidemiology (STROBE) guidelines (18). Data were collected from the target population between July and August 2021.

### Setting

This study was set up in Ethiopia. Ethiopia has 11 administrative regions and two city states. Four administrative regions were randomly selected for the study; namely Amhara, Oromia, Somali, and Southwest. See Supplementary Material 1 for sampling frame.

### Population

The population of interest was healthcare workers.

### Study size

Using a single proportion population formula, local or systemic side effect of Oxford AstraZeneca COVID-19 vaccine as an outcome measure, assuming that 50% of healthcare workers experience at least one side effect, 95% confidence level, and type I error of 5%, the sample size was 384.

The inclusion criterion was all healthcare workers who took at least one dose of the Oxford AstraZeneca COVID-19 vaccine because it was the main vaccine administered to healthcare workers during the first phase of the vaccination campaign in Ethiopia.

### Sampling

The sample size was distributed across the four regions. One healthcare institution was selected from each region because of the assumption that the type of healthcare institution will not influence the side effects and that the characteristics of healthcare workers among the healthcare institutions within a region were similar. Then, a list of vaccinated individuals from that healthcare institution was obtained. Finally, a random sample was taken from each list using MS Excel 2010 random number generator in Windows 10 operating system.

### Instrument

Data collection tool had been developed according to COVID-19 Vaccine Safety Tracking (CoVaST) methodology; and it had general demographic characteristics, medical anamnesis, COVID-19 Related anamnesis, and vaccine related anamnesis (19). The tool was pre-tested among 10 healthcare workers who took the Oxford AstraZeneca COVID-19 vaccine and were not part of the sample. Then, the tool was revised, for example by removing repeated question and re-writing a few questions for clarity. Please see Supplementary Material 2 for the tool. Data were collected by telephone interview.

Variables measured in this study were mentioned below:

- Predictor: Body Mass Index (BMI) (underweight, normal, overweight, and obese), presence of at least one chronic disease (yes vs. no), at least one medication currently taken by the patient (yes vs. no), diagnosis with COVID-19 (yes vs. no), and presence of COVID-19 like symptoms (yes vs. no). The source was the healthcare worker who responded to the study.
- Confounders: age (≤ 30 vs. >30), and sex (female vs. male) according to the healthcare worker
- Outcomes: the development of at least one local side effect (yes vs. no), and the development of at least one systemic side effect (yes vs. no).

### Ethics

The study was approved by the Institutional Review Board of Jimma University (IHRPG/320/21). Respondents provided informed verbal consent. Personal identifiers were not used to protect the confidentiality of the respondents. This study is important to improve clinical practice during vaccine delivery. Moreover, it is useful to improve national policy toward COVID-19 vaccines. These benefits were explained to the respondents.

### Analyses

Descriptive and inferential statistics were used. Test for confounders was done using *X*^2^ test and *t-*test depending on the type of variables. Moreover, *X*^2^ test was done to check correlation between predictors. Statistical significance was declared at *p*-value < 0.05. Finally, logistic regression was applied to measure the predictors of the outcome variables.

## Results

A total of 346 participated in the study with a response rate of 90.1%. All the 346 respondents took the first dose of the Oxford AstraZeneca COVID-19 vaccine.

### General characteristics of the respondents

One hundred and eighteen out of 346 (34.1%) respondents were female. The age of the respondents ranged from 20 to 62 with a mean of 31.0 and a standard deviation of 7.4.

Regarding the body mass index of the respondents, the majority of the respondents (*n* = 185 out of 282, 65.1%) had normal BMI. Only 2.1% of the respondents were obese. The mean BMI was 23.6 (SD = 3.0).

The most frequent profession of the respondents was nurse, 152 (43.70%). The least frequent were dentists (1), physiotherapists (1), and health educators (1).

**Figure 1:**
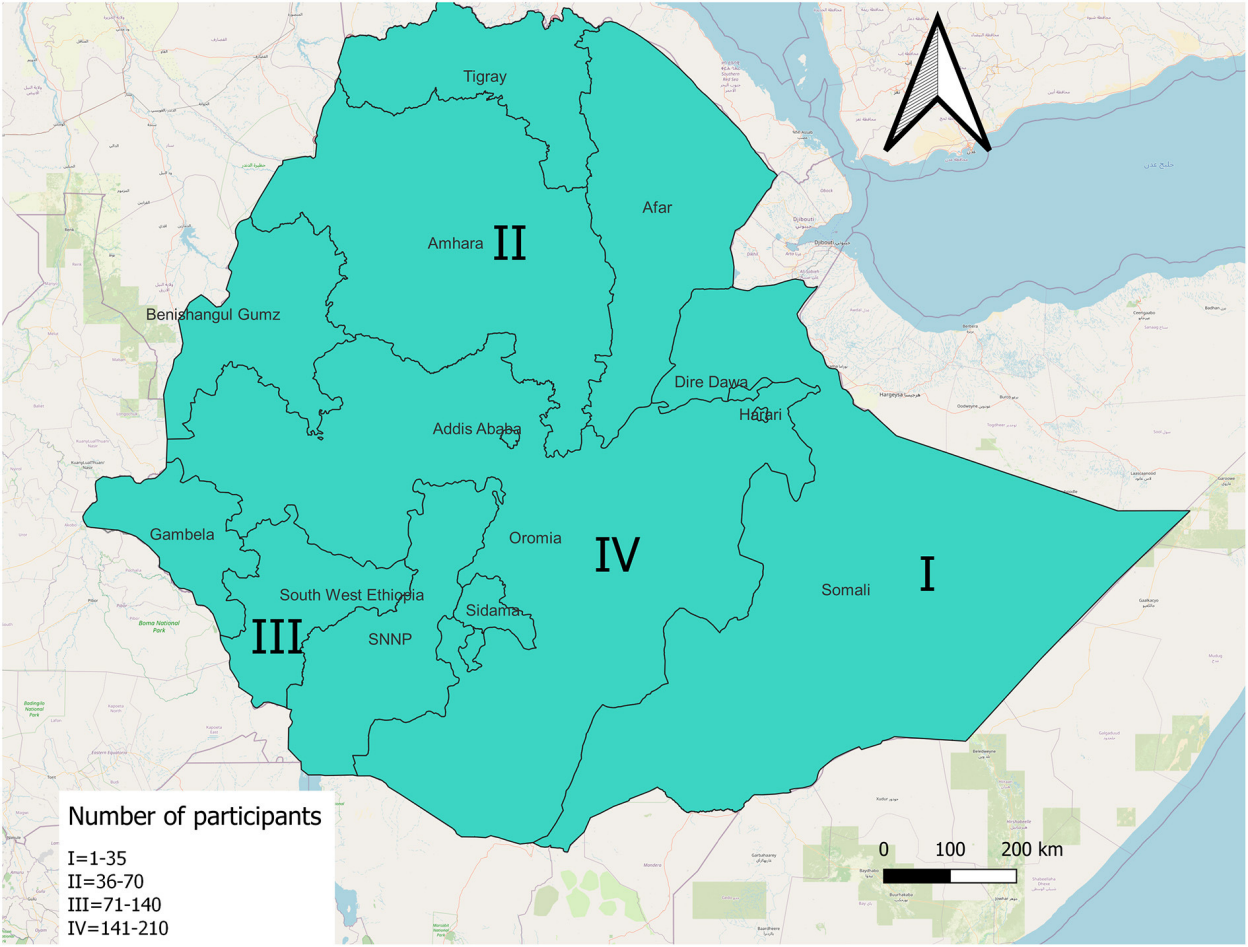
Study setting of self-reported side effects of COVID-19 vaccines, Ethiopia, 2021.

The most frequent number of responses, 149 out of 346 (43.1%) were from the Oromia region. The rest were from three other regions. See Figure 1. General characteristics of the respondents were described in Table 1.

**Table 1:** Distribution of characteristics on demography, profession, and region of respondents of self-reported side effects of COVID-19 vaccines, Ethiopia, 2021.

**Variable**	**Category**	**Frequency**	**Percentage**
Sex (*n* = 346)	Female	118	34.1
	Male	228	65.9
Age (*n* = 346)	≤ 30	230	66.5
	>30	116	33.5
Body mass index (*n* = 284)	Underweight	10	3.5
	Normal body weight	185	65.1
	Overweight	83	29.2
	Obese	6	2.1
Profession (*n* = 346)	Nurse	152	43.9
	Physician	124	35.8
	Health officer	15	4.3
	Laboratory technologist/technician	11	3.2
	Anesthetist	8	2.3
	Pharmacist	8	2.3
	Midwife	8	2.3
	Radiographer	6	1.7
	Other*	14	4.0
Region (*n* = 346)	Amhara	45	13.0
	Oromia	149	43.1
	Somali	32	9.2
	Southwest	120	34.7

*Other, environmental health (3), biomedical (3), public health (3), health informatics (2), dentist (1), physiotherapist (1), health education (1).

### Medical anamnesis

Thirty six out of 346 respondents (10.4%) reported at least one chronic disease. asthma (8), allergy (7), hypertensive heart disease (7), and type II diabetes mellitus (5) were frequently reported by the respondents. Fifty two out of 346 (15.0%) of the respondents reported that they are taking at least one medication at the time of the study. contraceptive (27), anti-asthma (8), anti-hypertension (6), and antidiabetic (6) drugs were frequently taken. see table 2.

**Table 2:** Chronic diseases and medications taken by the respondents of COVID-19 vaccine side effects, Ethiopia, 2021.

**Chronic disease (*****n*** = **346)**	**Frequency**	**Percentage**
Asthma	8	2.3
Allergy	7	2.0
Hypertensive heart disease	7	2.0
Type II diabetes mellitus	5	1.4
Renal disease	3	0.9
Other*	6	1.7
**Medication (*n* = 346)**		
Contraceptive	27	7.8
Anti-asthma	8	2.3
Antihypertensive	6	1.7
Antidiabetic	6	1.7
Antireflux	2	0.6
HAART**	2	0.6
Antibiotic	1	0.3

*Other, HIV/ AIDS (2), Deep Venous Thrombosis (1), cardiac disease (1), hepatologic disease (1), neurologic disease (1).

**HAART, Highly Active AntiRetroviral Therapy.

All of the respondents, 346 out of 346 (100.00%) reported to not smoke cigarettes.

Forty-three out of 346 (12.4%) of the respondents reported to drink alcohol. Forty-one respondents reported drinking beer and two reported wine. The number of glasses of 0.5 liter beer consumed per week per individual ranged from one to 98 (mean = 6.4, SD = 15.1).

Five out of 118 (4.2%) females and 18 out of 118 (15.3%) females were pregnant and breastfeeding at the time of vaccination, respectively.

More female (28.0%) compared to male (7.0%) reported to take at least one medication (*p*-value < 0.0001). See Table 3 for a description of medical anamnesis by sex.

**Table 3:** Medical anamnesis by sex of the respondents of COVID-19 vaccine side effects, Ethiopia, 2021.

**Variable**	**Number**	**Sex** **Frequency (percent)** **Female**	**Male**	**Sig**.*
Had chronic disease	346	13 (11.2)	21 (9.2)	0.593
Took medication	346	33 (28.0)	16 (7.0)	<0.0001
Mean alcohol consumption	41	2.0	8.1	0.258

*Significance values are two-sided and were based on x^2^ test for chronic disease and medication, and two-sample t-test for mean 0.5 liter beer consumed per week. Bold value of the Sig. column indicates statistical significance.

The prevalence of chronic diseases was more among respondents older than 30 years of age (16.4%) compared with respondents who were at least 30 years of age (*p*-value = 0.004). See Table 4.

**Table 4:** Medical anamnesis by age of the respondents of COVID-19 vaccine side effects, Ethiopia, 2021.

**Variable**	**Number**	**Age** **frequency (percent)** ≤ **30**	>**30**	**Sig**.*
Had chronic disease	346	15 (6.5)	19 (16.4)	0.004
Took medication	346	31 (13.5)	18 (15.5)	0.608
Mean alcohol consumption	41	8.2	5.2	0.541

*Significance values are two-sided and were based on x^2^ test for chronic disease and medication, and two-sample t-test for mean 0.5 liter beer consumed per week. Bold value of the Sig. column indicates statistical significance.

### COVID-19 related anamnesis

Thirteen out of 346 (3.8%) respondents have been diagnosed with COVID-19. 11 out of 13 (84.62%) of these were diagnosed before vaccination and the rest two were diagnosed after vaccination. Moreover, nine out of 13 (69.20%) had at least one mild symptom and the rest four (30.8%) had at least one moderate symptom. Five out of nine (55.6%) respondents had fever. Cough and headache each accounted for four out of nine (44.4%) respondents. Difficulty breathing, fatigue, and new loss of smell were each reported by three out of nine (33.3%) respondents. muscle ache, sore throat, and runny nose were each experienced by two out of nine (22.2%) respondents.

Sixty-one out of 346 (17.6%) respondents reported at least one COVID-19 like symptom even though they had never been diagnosed with COVID-19. Fever, cough, headache, fatigue, and muscle ache were the commonest symptoms. see Table 5.

**Table 5:** Distribution of COVID-19 like symptoms among the respondents who reported COVID-19 like symptoms of respondents of side effects of COVID-19 vaccine, Ethiopia, 2021.

**Symptom (*****n*** = **52)**	**Frequency**	**Percent**
Fever or chills	42	80.8
Cough	37	71.2
Headache	29	55.8
Fatigue	27	51.9
Muscle or body aches	24	46.2
New loss of taste or smell	12	23.1
Shortness of breath or difficulty breathing	7	13.5
Sore throat	6	11.5
Congestive or runny nose	3	5.8
Loss of appetite	2	3.8
Nausea or vomiting	1	1.9

respondents' experience of COVID-19 symptoms without being diagnosed with COVID-19 ranged from 2 days to 30 days. the mean duration of symptoms in days was 6.4 (SD = 5.1)

Fifty-three out of 348 (15.20%) respondents were tested for antibodies of COVID-19. Twenty-two out of 53 (41.51%) were positive.

There was no statistically significant relationship between COVID-19 anamnesis, and sex (Table 6) and age (Table 7).

**Table 6:** Relationship between COVID-19 anamnesis and sex among respondents of self-reported side effects of COVID-19 vaccines, Ethiopia, 2021.

**Variable**	**Number**	**Sex** **frequency (percent)** **Female**	**Male**	**Sig**.*
Diagnosis with COVID-19	346	4 (3.4)	9 (3.9)	0.796
Ever had symptoms of COVID-19	333	18 (15.8)	43 (19.6)	0.389

*Significance values are two-sided and were based on x^2^ test.

**Table 7:** Relationship between COVID-19 anamnesis and age among respondents of self-reported side effects of COVID-19 vaccines, Ethiopia, 2021.

**Variable**	**Number**	**Age** **frequency (percent)** ≤ **30**	>**30**	**Sig**.*
Diagnosis with COVID-19	346	8 (3.5)	5 (4.3)	0.701
Ever had symptoms of COVID-19	333	39 (17.5)	22 (20.0)	0.577

*Significance values are two-sided and were based on x^2^ test.

### Vaccine-related anamnesis

Three hundred forty-six out of 346 (100.0%) took the first dose of the Oxford AstraZeneca COVID-19 vaccine, 137 out of 346 (39.6%) took the second dose of the vaccine after 2 month.

One hundred and seventy five out of 346 (50.6%) respondents experienced at least one local side effect within 4 weeks of taking the vaccine. The most frequent local side effect was injection site pain experienced by 173 out of 346 (50.0%) respondents. see Table 8.

**Table 8:** Local side effects of COVID-19 vaccine among healthcare workers in Ethiopia, 2021.

**Local side effect (*****n*** = **346)**	**Frequency**	**Percent**
Injection site pain	173	50.0
Injection site swelling	4	1.2
Injection site redness	4	1.2
Itching	2	0.6

Local side effects were observed after the first dose only among the solid majority of the respondents with local side effects, 156 out of 175 (89.1%), after the second dose among three out of 175 (1.7%), and after both doses in 16 out of 175 (9.1%).

The duration of the local side effects were more frequent during the first, second, and third day after vaccination waning after day three. See Table 9.

**Table 9:** Duration of local side effects of COVID-19 vaccine among healthcare workers in Ethiopia, 2021.

**Duration of local side effects (*****n*** = **175)**	**Frequency**	**Percent**
1 day	59	33.7
2 day	54	30.9
3 days	42	24.0
5 days	13	7.4
1 week	5	2.9
2 weeks	1	0.6
4 weeks	1	0.6

One-hundred and fifty four out of 346 (44.5%) experienced at least one systemic side effect within 4 weeks of taking the COVID-19 vaccine. Headache, fever, and fatigue were frequent systemic side effects. see Table 10.

**Table 10:** Systemic side effects of COVID-19 vaccines among healthcare workers in Ethiopia, 2021.

**Systemic side effect (*****n*** = **346)**	**Frequency**	**Percent**
Headache	87	25.1
Fatigue	68	19.7
Fever	68	19.7
Joint pain	55	15.9
Muscle pain	50	14.5
Chills	31	9.0
Nausea	7	2.0
Change of taste	3	0.9
Loss of appetite	3	0.9
other *	8	2.3

*Other, Oral ulcers, blisters, vesicles (1), Skin rash (1), blurring of vision (1), cough (1), diaphoresis (1), rhinorrhea (1), vertigo (1), vomiting (1).

The systemic side effects emerged mostly after the first dose only, 136 out of 154 (88.3%), after the second dose only, 10 out of 154 (6.5%), and after both doses, eight out of 154 (5.2%).

The one day and 2 days duration of systemic side effects were frequent. See Table 11.

**Table 11:** Duration of systemic side effects of COVID-19 vaccines among healthcare workers in Ethiopia, 2021.

**Duration of systemic side effects (*****n*** = **154)**	**Frequency**	**Percent**
1 day	51	33.1
2 day	51	30.1
3 days	31	20.1
5 days	12	7.8
1 week	6	3.9
2 weeks	3	1.9

Fifty three out of 154 (34.4%) took at least one medication to relieve the side effect. Paracetamol, 30 out of 50 (60.0%) and diclofenac sodium, 11 out of 50 (22.0%) were the most frequently taken medications to relieve the side effects. One healthcare worker with chills was administered with ceftriaxone. see Table 12 on the medications taken by the respondents to relieve side effects.

**Table 12:** Medications taken by healthcare workers to relieve the side effects of COVID-19 vaccines, Ethiopia.

**Medication to relieve side effects (*****n*** = **50)**	**Frequency**	**Percent**
Paracetamol	30	60.0
Diclofenac sodium	11	22.0
Ibuprofen	8	16.0
		
Ceftriaxone	1	2.0

Forty two out of 51 (82.4%) took the medications after the vaccination. the remaining nine (17.6%) took the medications before the vaccination.

### Predictors of Side Effects

#### Correlation test

Chronic disease and taking medication were not independent. 24 out of 34 (70.6%) with chronic disease took medication while only 25 out of 312 (8.0%) without chronic disease took medication. This was statistically significant using an *x*^2^ test (*p*-value < 0.0001).

Using independent sample *t-*test, the mean number of 0.5 liter beer consumed per week and chronic disease are not statistically significantly associated, *p*-value = 0.788.

#### Predictors of local side effect

There was no a statistically significant association between COVID-19 like symptoms and local side effects of the Oxford AstraZeneca COVID-19 vaccine after controlling for age and chronic diseases. See Table 13.

**Table 13:** Binary logistic regression model, the association between COVID-19 like symptom and local side effect among healthcare workers in Ethiopia, 2021.

**Variable**	**Response**	**Adjusted OR**	**Sig**
COVID-19 like symptom*	Yes	0.8 (0.5, 1.3)	0.582
	No	1	
Age	≤ 30	1.1 (0.7, 1.7)	0.611
	>30	1	
Chronic disease	Yes	1.2 (0.6, 2.3)	0.606
	No		

*controlling for age and chronic disease.

#### Predictors of systemic side effect

There was a statistically significant association between COVID-19 like symptoms and systemic side effects of the Oxford AstraZeneca COVID-19 vaccine after controlling for age and chronic diseases. See Table 14.

**Table 14:** Binary logistic regression model, the association between COVID-19 like symptom and systemic side effect among healthcare workers in Ethiopia, 2021.

**Variable**	**Response**	**Adjusted OR**	**Sig**
COVID-19 like symptom*	Yes	1.38 (1.04, 1.82)	0.025
	No	1	
Age	≤ 30	0.86 (0.68, 1.09)	0.204
	>30	1	
Chronic disease	Yes	1.37 (0.93, 2.00)	0.108
	No	1	

*controlling for age and chronic disease. Bold value of the Sig. column indicates statistical significance.

## Discussion

The results of our study showed that all the respondents have taken the Oxford AstraZeneca COVID-19 vaccine at least once. Nearly 40% took the second dose. Slightly above 50% of the respondents experienced local side effects of the Oxford AstraZeneca COVID-19 vaccine, the major local side effect being injection site pain experienced by 50% of all the respondents. It commonly occurs after the first dose and lasts mostly until the third day after vaccination.

Like the local side effects, systemic side effects were experienced by nearly 44.5% of the respondents. Headache, fatigue, fever, joint pain, muscle pain, and chills are the most common systemic side effects of the Oxford AstraZeneca COVID-19 vaccine. They are commonly observed after the first dose and they usually lasted for 1 to 3 days waning afterwards. Paracetamol, diclofenac sodium, and ibuprofen are commonly used after systemic side effects.

Studies elsewhere in Africa, such as Ghana reported higher rates of at least one side effect (81%) among healthcare workers (20). Nonetheless, the prevalenve of headache and fever among healthcare workers after receiving the Oxford AstraZeneca COVID-19 vaccine in Ethiopia is similar with Ghana. Contrary to Ethiopia, Egyptian healthcare workers reported a higher rate of fatigue, 20 vs. 57% and headache, 25 vs. 50% (21). The reasons for the differences in systemic side effects between healthcare workers in Ethiopia and Egypt are unclear.

The prevalence of headache, fever and muscle ache in this study is similar with a study from the Czech Republic and Slovakia (22, 23). However, injection site pain, fatigue and chills after vaccination are much lower in our study. This might be due to the type of vaccine (24). Pfizer–BioNTech COVID-19 vaccine was used in both the Czech Republic and Slovakia, in Ethiopia Oxford AstraZeneca vaccine was used. Duration of symptoms in our study is also similar with studies from the Czech Republic and Slovakia, the symptoms mostly lasting between the first and the third day after vaccination (22, 23).

A study from Jordan which mainly used vaccines Oxford AstraZeneca and Pfizer–BioNTech also reported a similar prevalence of headache, fever, and muscle pain (25). However, it reported more fatigue. Even though the mean age of the participants from the Czech Republic (42.6) and Slovakia (37.8) were older than in Ethiopia (31.0), the mean age from Jordan (35.0) is similar. Therefore, age might not explain the differences in the prevalence of fatigue.

Antihistamines were commonly used in the Czech Republic (23), but analgesics and non-steroidal anti-inflammatory drugs were commonly used in Ethiopia. Similarly, in Togo analgesics were commonly used (26).

There is no a statistically significant predictor of local side effects.

After controlling for confounders, such as age and chronic diseases, presence of COVID-19 like symptom is the only statistically significant predictor of systemic side effects of the Oxford AstraZeneca COVID-19 vaccine.

### Policy and practice implications

Physicians might help by counseling the clients with the fact that even though local side effects are common, systemic side effects are less common and that they subside within a day, two, or three. Moreover, physicians and pharmacists can help in monitoring the doses of paracetamol and diclofenac sodium taken by people to relieve side effects so as to prevent toxicity.

Policy wise, the Ministry of Health should consider the presence of COVID-19 symptoms before giving vaccines. It may revise the guidelines to instruct vaccine providers that healthcare workers wait for their COVID-19 like symptoms to subside before taking the vaccines.

This study is strong in that we took sample of healthcare workers across four regions of Ethiopia.

We did not measure psychological factors reported as predictors of side effects by other study. This might limit the findings of the study. Moreover, the design is a cross-sectional study which limits the ability to establish temporality between the predictors and the outcome measure, and suffers from recall bias. Finally, non-response rate and design effect were not considered during sample size calculation. These also limit the findings of the study.

## Conclusion

The prevalence of local- and systemic-side effects of the Oxford AstraZeneca vaccine was modest. As the symptoms were mostly common in the first 3 days, it is preferable to monitor healthcare workers at least in the first 3 days following the administration of the vaccine. Moreover, physicians and pharmacists should monitor the use of paracetamol and diclofenac sodium. Vaccination guidelines by the Ministry of Health should consider COVID-19 like symptoms before the provision of the COVID-19 vaccine to an individual.

## Data availability statement

The raw data supporting the conclusions of this article will be made available by the authors, without undue reservation.

## Ethics statement

The studies involving human participants were reviewed and approved by Institutional Review Board of Jimma University. Written informed consent for participation was not required for this study in accordance with the National Legislation and the Institutional requirements.

## Author contributions

EY: conception, design, data collection, data analysis, first draft writing, and final draft writing. AR and MK: conception, design, data collection, and final draft review. AS-M, MS, AM, SE, FM, SM, BA, MY, AU, and JA: design, data collection, and final draft review. All authors contributed to the article and approved the submitted version.

## Conflict of interest

The authors declare that the research was conducted in the absence of any commercial or financial relationships that could be construed as a potential conflict of interest.

## Publisher's note

All claims expressed in this article are solely those of the authors and do not necessarily represent those of their affiliated organizations, or those of the publisher, the editors and the reviewers. Any product that may be evaluated in this article, or claim that may be made by its manufacturer, is not guaranteed or endorsed by the publisher.
