# Supplementary material for: Self-reported side effects of the Oxford AstraZeneca COVID-19 vaccine among healthcare workers in Ethiopia, Africa: A cross-sectional study
Source: Front Public Health. 2022 Jul 19;10:937794. doi: 10.3389/fpubh.2022.937794 (PMC9343757; doi:10.3389/fpubh.2022.937794)
Supplement: Supplementary file 1 [file Data_Sheet_1.pdf]

Self-reported Side Effects of COVID-19 Vaccines among Healthcare Workers in Ethiopia, Africa: a cross-sectional study

Supplementary material

Supplementary material 1: Sampling frame

Region	Total number of health professionals	Number selected
Oromia	54406	149
Amhara	40375	45
Somali	9982	32
Southwest	7694	120

Supplementary material 2: Tool

**1. Demographic Data**	
1.1 Gender	1.Female<br>2.Male<br>3.Prefer not to say
1.2 Age(18-99)	
1.3 Profession	1.Physician (M.D.)<br>2.Dentist (D.D.S)<br>3.Nurse (R.N.)<br>4.MIDWIFE<br>5.Pharmacist (PharmD)<br>6.Physiotherapist<br>7.Other (please specify)
1.4 COUNTRY	• ETHIOPIA
1.5.Region	1.AMHARA<br>2.OROMIA<br>3.SOMALI<br>4.SOUTHWEST
1.6.Weight(kg)	
1.7.Height(meter)	
**2. Medical Anamnesis**	
2.1 Do you have any chronic disease?	1.Yes<br>0.No
2.2 If "Yes", please specify all chronic diseases you suffer from currently	1.Allergy<br>2.Asthma<br>3.Blood Disease<br>4.Bowel Disease<br>5.Cancer<br>6.Cardiac Disease<br>7.Chronic Hypertension<br>8.COPD<br>9.Diabetes Mellitus – I<br>10.Diabetes Mellitus – II<br>11.Hepatologic Disease<br>12.Psychological Distress<br>13.Neurologic Disease<br>14.Ophthalmologic Disease<br>15.Renal Disease<br>16.Rheumatoid Arthritis

	17.Thyroid Disease<br>18.Other, please specify
2.3 Do you take any medication currently?	1.Yes<br>0.No
2.4 If "Yes", please specify the category of the drug	1.Anti-asthma<br>2.Antibiotics<br>3.Anticoagulant<br>4.Antidepressant<br>5.Antidiabetic<br>6.Antiepileptic<br>7.Antihistamine<br>8.Antihypertensive<br>9.Anti-Reflux<br>10.Anti-venous Insufficiency<br>11.Immunosuppressive<br>12.Cholesterol-lowering<br>13.Common Analgesic<br>14.Contraceptive<br>15.Corticosteroid<br>16.NSAID<br>17.Opioid Analgesic<br>18.Thyroid Hormones<br>19.Other, please specify (the generic name or the market name of the drug)
2.5 Do you smoke cigarettes?	1.Yes<br>0.No
2.6 If "Yes", how many cigarettes do you smoke per day?	
2.7 Do you drink alcohol?	1.Yes<br>0.No
2.8 If "Yes", how many glasses of (0.5 l) beer per week?	
2.9. If "Yes", how many glasses of (0.2 l) Wine per week?	
2.10 If "Yes", how many glasses of (0.04 l) Spirit per week?	
2.11Were you pregnant during vaccination?(only for female)	1.Yes<br>0.no
2.12 Were you breast feeding during vaccination?(only for female)	1.Yes<br>0.no
**3. COVID-19-relate**	

3.1 Vaccine Type	1. Oxford–AstraZenecaCOVID-19 Vaccine(covishield)<br>2. Pfizer-BioNTech COVID-19 Vaccine<br>3. Moderna COVID-19 Vaccine<br>4. Janssen Vaccine<br>5. Sputnik V Vaccine<br>6. synovax<br>7. Covaxin Vaccine<br>8. Other, please specify
3.2 Vaccination Date (first dose)	
3.3 Have you taken the second dose?	1. Yes<br>0. No
3.4 Vaccination Date (second dose)	
3.5 Have you ever been diagnosed with COVID-19?	1.Yes<br>0.No
3.6 If "Yes", when were you diagnosed?	1. Before vaccination<br>2. Between 1st and 2nd dose of vaccine<br>3. After second dose
3.7. .Please specify the date when you were diagnosed	
3.8 How do you describe the severity of your COVID-19 infection?	1. **Mild** (no symptoms, or mild upper respiratory tract symptoms, or cough, new myalgia, or asthenia without new shortness of breath or a reduction in oxygen saturation)<br>2. **Moderate** (prostration, severe asthenia, fever > 38℃ or persistent cough clinical or radiological signs of lung involvement no clinical or laboratory indicators of clinical severity or respiratory impairment)<br>3. **Severe** (respiratory rate ≥ 30 breaths/min, or oxygen saturation ≤ 92% at a rest state, or arterial partial pressure of oxygen (PaO2)/ inspired oxygen fraction (FiO2) ≤ 300)<br>4. **Critical** (Respiratory failure Occurrence of severe respiratory failure (PaO2/FiO2 < 200), respiratory distress or acute respiratory distress syndrome (ARDS). This includes patients deteriorating despite advanced forms of respiratory support (non-invasive ventilation (NIV), high-flow nasal oxygen (HFNO)) OR patients requiring mechanical ventilation. OR other signs of significant deterioration hypotension or shock impairment of consciousness other organ failure)
3.9 What were the symptoms you have experienced during the COVID-19 infection?	1. Fever or chills<br>2. Cough<br>3. Shortness of breath or difficulty breathing<br>4. Fatigue<br>5. Muscle or body aches<br>6. Headache<br>7. New loss of taste or smell<br>8. Sore throat<br>9. Congestion or runny nose

	10. Nausea or vomiting<br>11. Diarrhea<br>12. Other (please specify)
3.10 For how many days did you experience the COVID-19 symptoms?	
3.11 If not diagnosed with covid 19, did you ever have the symptoms of covid 19?	1.Yes<br>• 0. no
3.12 If yes, What were the symptoms you have experienced?	1.Fever or chills<br>2.Cough<br>3.Shortness of breath or difficulty breathing<br>4.Fatigue<br>5.Muscle or body aches<br>6.Headache<br>7.New loss of taste or smell<br>8.Sore throat<br>9.Congestion or runny nose<br>10.Nausea or vomiting<br>11.Diarrhea<br>12.Other (please specify)
3.14 For how many days did you experience the COVID-19 symptoms?	
3.15 Have you ever tested for antibody of covid 19?	1.Yes<br>0.no
3.16 If yes, what was the result?	1.Positive<br>2.negative
**4. Vaccine Side Effects**	
4.1. Within four weeks of receiving the vaccine, have you suffered from any of the following local side effects?	1. Injection site pain<br>2. Injection site swelling<br>3. Injection site redness<br>4. Other, please specify
4.2. When did the local side effects emerge?	1. After the first dose only<br>2. After the second dose only<br>3. After both doses
4.3 If you chose any of the previous side effects, please	1. 1 day<br>2. 2 days

indicate their duration	3. 3 days<br>4. 5 days<br>5. 1 week<br>6. 2 weeks<br>7. 3 weeks<br>8. 4 weeks<br>9. > 1 month
4.4. Within four weeks of receiving the vaccine, have you suffered from any of the following side effects?	0. None<br>1. Fatigue<br>2. Headache<br>3. Muscle Pain<br>4. Joint Pain<br>5. Fever<br>6. Chills<br>7. Nausea<br>8. Diarrhoea<br>9. Shortness of breath<br>10. Anaphylaxis<br>11. Swollen lymph nodes<br>12. Mouth tingling<br>13. Loss of taste<br>14. Change of taste<br>15. Halitosis (Oral malodour)<br>16. Oral ulcers / blisters / vesicles<br>17. Bleeding gingiva<br>18. Skin rash<br>19. Other, please specify
4.5. When did the systemic side effects emerge?	1. After the first dose only<br>2. After the second dose only<br>3.After both doses
4.6. If you chose any of the previous side effects, please indicate their duration	1. 1 day<br>2. 2 days<br>3. 3 days<br>4. 5 days<br>5. 1 week<br>6. 2 weeks<br>7. 3 weeks<br>8. 4 weeks<br>9. > 1 month
4.7 Have you taken any medications to relieve your side effects?	1.Yes<br>0.No
4.8 If "Yes", please specify what drug you have used. Use either generic name or market name	

4.9 Was it before or after vaccination?	
4.10 Do you agree to participate in the longitudinal study evaluating the safety of the vaccines from a long term perspective?	1.Yes<br>0.No
4.11. If yes, give us please your contact e-mail address. Your e-mail address will be automatically stored and removed from the survey so the data you have shared will remain anonymous.	
